# Potential Benefits of Allogeneic Haploidentical Adipose Tissue-Derived Stromal Vascular Fraction in a Hutchinson–Gilford Progeria Syndrome Patient

**DOI:** 10.3389/fbioe.2020.574010

**Published:** 2020-10-21

**Authors:** Jaewoo Pak, Jung Hun Lee, Jeong Ho Jeon, Young Bae Kim, Byeong Chul Jeong, Sang Hee Lee

**Affiliations:** ^1^Mipro Medical Clinic, Seoul, South Korea; ^2^First Medical Center, Cerritos, CA, United States; ^3^National Leading Research Laboratory, Department of Biological Sciences, Myongji University, Yongin, South Korea; ^4^Biotechnology Program, North Shore Community College, Danvers, MA, United States

**Keywords:** Hutchinson–Gilford progeria syndrome, adipose tissue, stromal vascular fraction, progerin, insulin-like growth factor 1

## Abstract

Hutchinson–Gilford progeria syndrome (HGPS) is a rare, fatal, and genetic disorder in the *LMNA* gene encoding for prelamin A. Normally, prelamin A is processed to become lamin A protein. In HGPS patients, there is a heterozygous mutation in *LMNA* gene, in which there is a deletion of genetic codes responsible for 50 amino acids at the C-terminus of prelamin A. The processing of the abnormal prelamin A results in abnormal lamin A protein, called progerin, causing symptoms of accelerated early aging, probably due to the inflammaging process. It is well known that adipose tissue-derived mesenchymal stem cells (MSCs) have anti-inflammatory effects by modulating inflammatory cytokines and by extracellular vesicles. Here, we present a case of an HGPS patient who responded positively to injections of allogeneic haploidentical adipose tissue-derived stromal vascular fractions containing MSCs by showing rapid height and weight growth along with increased blood level of insulin-like growth factor 1.

## Background

**FIGURE 1:**
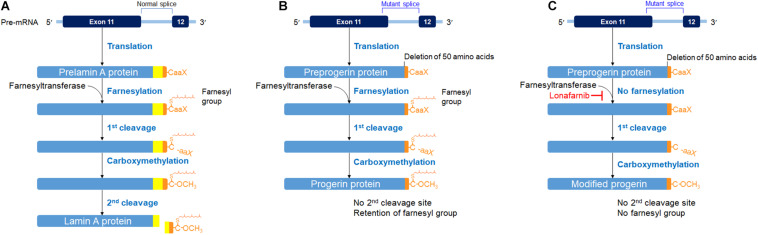
Posttranslational processing steps producing lamin A **(A)** in a normal cell, progerin **(B)** in an HGPS cell, and a modified progerin in a lonafarnib-treated HGPS cell **(C)**. A prelamin A polypeptide chain has its C-terminal – CaaX box (C, cysteine; aa, two aliphatic amino acids; X, any amino acid). The α-helical rod domain is divided into segments for displaying the progerin defect. The first cleavage was carried out by the zinc metalloprotease (Zmpste24) or Ras-converting enzyme (RCE1) and the second cleavage by Zmpste24. These figures were prepared using data adapted from Gordon et al. (2018).

Hutchinson–Gilford progeria syndrome (HGPS), also known as progeria, is a very rare, fatal genetic disorder caused by heterozygous mutation in the *LMNA* gene, which encodes for two nuclear membrane proteins, called lamins A and C. In normal conditions, *LMNA* gene produces prelamin A, which then undergoes a series of processing steps, including farnesylation of C terminus. Such farnesylation of the C-terminus is later removed off in the second cleavage of the processing, transforming prelamin A to lamin A (Figure 1A; Sinha et al., 2014; Harhouri et al., 2018). However, in HGPS genetic mutation, the *LMNA* gene codes responsible for the 50 amino acids near the C-terminus have been deleted, disrupting the second cleavage site. With the disruption of the cleavage site, the farnesyl group that is supposed to be removed off from the C-terminus remains. The abnormal farnesylated prelamin A, which is also called progerin, accumulates and becomes incorporated into the nuclear membrane lamina and exerts damage to cells as HGPS patients show an accelerated aging process (Sinha et al., 2014; Harhouri et al., 2018; Figure 1B). In HGPS cells, however, the wild-type *LMNA* gene is also expressed. Thus, a combination of lamin A/C and progerin is detectable at the nuclear level, although the molecular reason for such a phenomenon is not well understood (Yang et al., 2008; Piekarowicz et al., 2019). Because of the laminopathy created by progerin, HGPS patients show growth retardation early in their childhood, along with other symptoms of physiological aging: loss of hair, skin thinning, joint rigidity, osteoporosis, and so on. The average height growth rate in HGPS patients is approximately 3.5 cm per year, and the weight growth rate is approximately 0.5 kg per year during the ages of 1–10 years (Merideth et al., 2008). Their average age of survival is approximately 13.5–14.5 years, and the life expectancy ranges from 8 to 21 years, with cardiovascular diseases being the major cause of death (Sinha et al., 2014; Harhouri et al., 2018).

Recently, lonafarnib, a farnesyltransferase inhibitor, which is also used as a chemotherapeutic agent, has been tried to treat the HGPS patients, resulting in the possibility of improving the mortality rate of the patients by 1.6 years or more (Gordon et al., 2014, 2018). Lonafarnib blocks the farnesylation process of prelamin A (Gordon et al., 2018). The end protein is a modified progerin with shorter amino acids but without the farnesyl group attached (Figure 1C). In other words, the end product is a new product, identical neither to lamin A protein nor to progerin (Gordon et al., 2018). Consequently, taking lonafarnib is not free of side effects. Accumulation of non-farnesylated prelamin A has caused cardiomyopathy in a mouse model (Davies et al., 2010). Therefore, the side effects of taking the medication had been the major drawback for the HGPS patient being presented in this case report.

Mesenchymal stem cells (MSCs) are defined as multipotent stem cells that (i) can adhere to plastics; (ii) can express the surface molecules such as CD105 and CD73 and lack expression of CD45, CD34, CD14 or CD11b, CD79α or CD19, and HLA-DR; and (iii) can differentiate to osteocytes, adipocytes, and chondrocytes (Dominici et al., 2006). In 2001 and 2002, Zuk et al. (2002) showed that adipose tissue in the form of stromal vascular fraction (SVF) contains many multipotent stem cells that have the characteristics defined as MSCs. Mesenchymal stem cells, or adipose tissue-derived mesenchymal stromal cells, are known to exert anti-inflammatory effects and produce a large amount of exosomes that are rich in active enzymes, proteins, and other cytoplasmic/nuclear constituents (Arslan et al., 2013; Lai et al., 2013a,b). Adipose tissue-derived stem cells (ASCs) are one type of MSCs that can be obtained through manufacturing adipose SVF. When adipose SVF was used in the fat transplant, there was decreased excretion of interleukin 6 (IL-6) and tumor necrosis factor α, which are proinflammatory cytokines and increase excretion of IL-10, an anti-inflammatory cytokine (Zhu et al., 2019). Such adipose SVF has been used in human patients since 2009 by injecting intra-articularly and intravenously without any serious side effects reported (Pak et al., 2013; Berman and Lander, 2017).

It has also been reported that extracellular vesicles (EVs), including exosomes, released from MSCs are shown to have anti-inflammatory and immunomodulatory effects (Del Fattore et al., 2015; Börger et al., 2017; Chang et al., 2018; Deng et al., 2019).

It has been shown in an animal study that injection of EVs derived from the serum of young mice to the old mice may have rejuvenated the aged T cells, possibly attenuating the “inflammaging” process (Wang et al., 2018).

Here, we present a case of one HGPS patient who received allogeneic, haploidentical adipose tissue-derived SVF containing MSCs and showed relatively significant growth in height and weight, along with about 50% increase in his plasma insulin-like growth factor 1 (IGF-1) level.

## Case Presentation

### Patient

The patient is a 12.8-year-old male with HGPS diagnosed in early childhood. He had previously tried lonafarnib for about 7 years from 2010 to 2017. He decided to discontinue the medication because of side effects of skin changes; chronic nausea, which became very severe while traveling (the patient temporarily quit the medication while traveling); and loss of energy described as a weakness. For about 2 years before visiting the Mipro Medical Clinic in July 2019, the patient was only on vitamin D and liver vitamins containing riboflavin 5 mg, thiamine 10 mg, and ursodeoxycholic acid 50 mg for liver and for the chronic elevation of liver enzymes.

This case report complies with the Declaration of Helsinki. Further, in 2009, the Korean Food and Drug Administration has allowed the uses of adipose SVF (not the expanded and cultured ASCs) for medical treatments (Korean Food and Drug Administration (KFDA), 2009). Informed consent was obtained from the patient’s guardian, accordingly.

The inclusion criteria, exclusion criteria, and outcome endpoints were listed as follows. The inclusion criteria were (i) diagnosis of HGPS, (ii) males or females, (iii) age over 10, and (iv) unwillingness to proceed with lonafarnib medication. Exclusion criteria were (i) concomitant connective tissue disease thought to impact the HGPS (i.e., lupus, rheumatoid arthritis, and fibromyalgia); (ii) concomitant endocrine disorder that might impact results (i.e., hypothyroidism and diabetes); (iii) concomitant neurologic disorder that might impact results (i.e., peripheral neuropathy and multiple sclerosis); (iv) active cardiac disease; and (v) active pulmonary disease requiring medication usage.

Outcome endpoints (obtained 12, 44, 62, and 134 days after the completion of treatments) were (i) pretreatment and posttreatment height, (ii) pretreatment and posttreatment weight, and (iii) pretreatment and posttreatment IGF-1 levels.

For 2 weeks before the procedure, the patient was restricted from taking any steroids and other herb medications.

### Liposuction, Isolation of Adipose SVF, and Adipose SVF Injection

**FIGURE 2:**
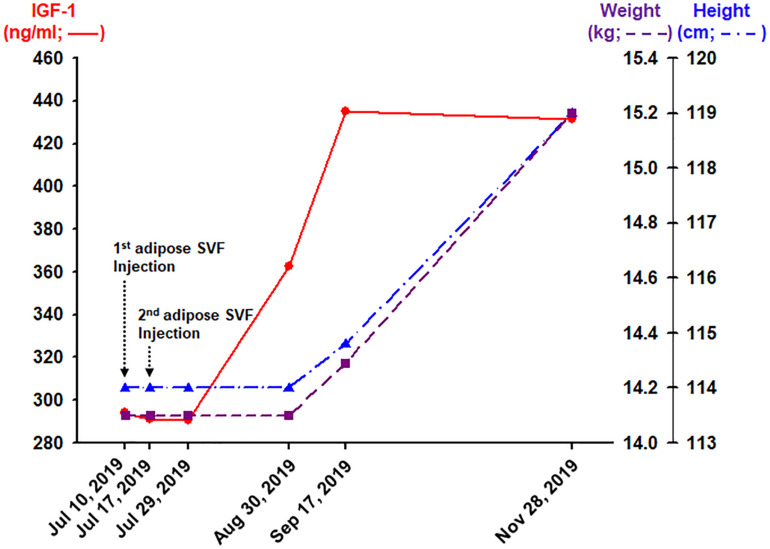
Outcomes obtained 12, 44, 62, and 134 days after the completion (the second adipose SVF injection) of treatment of the HGPS patient.

After typing and matching, the donor (in this case, the patient’s mother) went through two separate occasions of liposuction, 1 week apart. The detailed description of the procedure describing the liposuction and isolation of adipose SVF was described in a previous report (Pak et al., 2018). Briefly, in the operating room, 200 g of subcutaneous adipose tissue was obtained from the lower abdominal area using a clean, sterile technique on July 10, 2019, and another 200 g from the upper abdomen area 1 week later, on July 17. The adipose tissue was then processed as previously described (Pak et al., 2018). At the end of the procedure, about 10 mL of adipose SVF was isolated on each occasion. The isolated adipose SVF was then filtered three times using 100-μm metal filters (Pak et al., 2018), to remove any large debris. Afterward, the 10-mL filtered adipose SVF was added with 10 mL of normal saline solution. The total of 20 mL allogeneic haploidentical adipose SVF was then injected into the patient as a slow intravenous push (IVP) over 10 min. Total, two IVP injections of adipose SVF were performed on two separate occasions, 1 week apart (Figure 2).

## Description of Clinical Outcomes

About 62 days after the second IVP injection of allogeneic, haploidentical adipose SVF, the patient’s blood IGF-1 level increased approximately 50% from 294.0 to 434.9 ng/mL (Figure 2). Consequently, 134 days after the second SVF injection, the HGPS patient noted a 5-cm growth in height and 1.1 kg of weight gain (Figure 2). Furthermore, the patient noticed an improved sense of well-being with increased energy, appetite, and food intake. No side effect has been reported.

## Discussion

Other than IGF-1 levels and liver function test, the available patient’s blood results did not show much change from July 2019 to November 2019. The patient has been having a chronic elevation of liver function test results, including alanine aminotransferase (ALT), which was reported to be 140 U/L on the prior blood test. For such abnormality, he has been taking the liver vitamins containing ursodeoxycholic acid. The patient discontinued the liver vitamins a few days before the first injection of the adipose SVF. Because the patient previously had abnormally elevated blood liver function test results, the discontinuation of the liver vitamins may probably have caused the elevation of ALT/aspartate aminotransferase and other liver enzymes, instead of the injections of adipose SVF.

Hutchinson–Gilford progeria syndrome patients have growth retardation beginning in their childhood due to the laminopathy caused by progerin, which is also shown to accumulate in normal aging human patients (Scaffidi and Misteli, 2006). The average rate of height growth in HGPS patients is about 3.5 cm per year during the ages of 1–10 years. At around 13 years old, HGPS patients start to have cardiovascular problems, which is comparable to the age of 60s in the general normal population (Sinha et al., 2014). The average age of survival in HGPS patients is approximately 14 years. Thus, the height increase of 5 cm over 4 months in this 13-year-old HGPS patient (Figure 2) may be equivalent to a growth spurt in a patient in his 60s, which may be considered to be highly unexpected.

Along with the height increase, the patient gained more than 1 kg over the same period (Figure 2). This weight gain may be significant because the average weight gain in HGPS patients is about 0.5 kg per year. Weights can be variable during certain times of the day, depending on oral intakes and bodily secretions. Thus, to ensure the minimal true weight as possible, the weight was measured in the morning, right after waking up and emptying his bladder. Because the other earlier weight measurements were performed after meals in the morning, the chance of the patient weight gain being a daily variation is very unlikely.

Along with the height increase, there was an almost 50% increase in the blood level of IGF-1 (Figure 2). The steep rise of IGF-1 is highly associated with a growth spurt during puberty (Cole et al., 2015). This HGPS patient being 13 years old, having such puberty-like changes during the time of high morbidity rate in the HGPS patient population, may signify that there may be some positive changes at the cellular level. In addition, IGF-1 has been shown to be increased in animal studies to have longevity effects (Marino et al., 2010). Likewise, the increase in IGF-1 may signify the possibility of extending longevity in this HGPS patient.

It is very well known that mammalian cells naturally release EVs containing various bioactive materials including fragments of DNAs, RNAs, enzymes, proteins, lipids, mitochondria, and other cellular materials and organelles (Gurunathan et al., 2019). Among the three different types of EVs (apoptotic bodies, exosomes, and microvesicles), most data are available with regard to exosomes (Gurunathan et al., 2019; Elahi et al., 2020). After being originated from the endosomal system of each cell, exosomes are released into the extracellular space (Raposo and Stoorvogel, 2013). While in the extracellular space, exosomes are internalized by host cells via a mechanism involving the fusion of the cell membranes or by phagocytosis. Once internalized, exosomes release their contents into the recipient cells, potentially exerting regenerative or rejuvenating effects by improving or replacing the needed cellular cytoplasmic/nuclear contents (Raposo and Stoorvogel, 2013).

MSCs are well known to release a large number of EVs, including exosomes (Elahi et al., 2020). Also, it is well known that MSCs have anti-inflammatory effects via a paracrine mechanism. In HGPS patients, when intact donor MSCs are injected intravenously, some of the cells may pass through the pulmonary vasculature and potentially replenish the mesoderm-derived tissues with new functional multipotent stem cells to help maintain the homeostasis (Park et al., 2018; Kallmeyer et al., 2020). This would be another potential mechanism explaining the positive effects shown in this patient.

To the best of our knowledge, this is the first case report showing only the possibility of potential positive effects of allogeneic haploidentical transplants of adipose SVF containing MSCs in a patient with a fatal genetic disorder. Adipose tissue-derived stromal cells have been used in human patients intravenously and intra-articularly since 2009 without any major side effects. With the possibility of MSCs providing the anti-inflammaging effects, applying allogeneic haploidentical adipose SVF can be a rudimental but potentially a dramatic treatment modality for, possibly, fatal genetic diseases and other diseases that involve inflammaging process. As a study in the future, it would be better to check the phenotype of the nucleus of the cells in HGPS patients after applying allogeneic haploidentical adipose SVF to see if the nuclear membrane has returned to a round shape without any blebs. With the confirmation, larger studies with more participants would be necessary to assess the true potential benefits of adipose SVF in HGPS patients and other similar fatal genetic disorders.

## Data Availability Statement

All datasets presented in this study are included in the article/supplementary material.

## Ethics Statement

The studies involving human participants were reviewed and approved by Mipro Medical Clinic Institutional Review Board committee (MMCIRB). Written informed consent to participate in this study was provided by the participants’ legal guardian/next of kin. Written informed consent was obtained from the individual(s), and minor(s)’ legal guardian/next of kin, for the publication of any potentially identifiable images or data included in this article.

## Author Contributions

JP, JHL, and SHL equally contributed to writing the manuscript and collecting data. JP and JHJ were major contributors in collecting data and writing the manuscript. JP, YBK, and BCJ participated in the interpretation of data and contributed to the writing of the manuscript. All authors read and approved the final manuscript.

## Conflict of Interest

The authors declare that the research was conducted in the absence of any commercial or financial relationships that could be construed as a potential conflict of interest.
